# Supplementary figures and images for: Transcriptomic profiling of pancreatic neuroendocrine tumors: dysregulation of WNT, MAPK, PI3K, neddylation pathways and potential non-invasive biomarkers
Source: PLoS One. 2025 Jun 16;20(6):e0325672. doi: 10.1371/journal.pone.0325672 (PMC12169574; doi:10.1371/journal.pone.0325672)

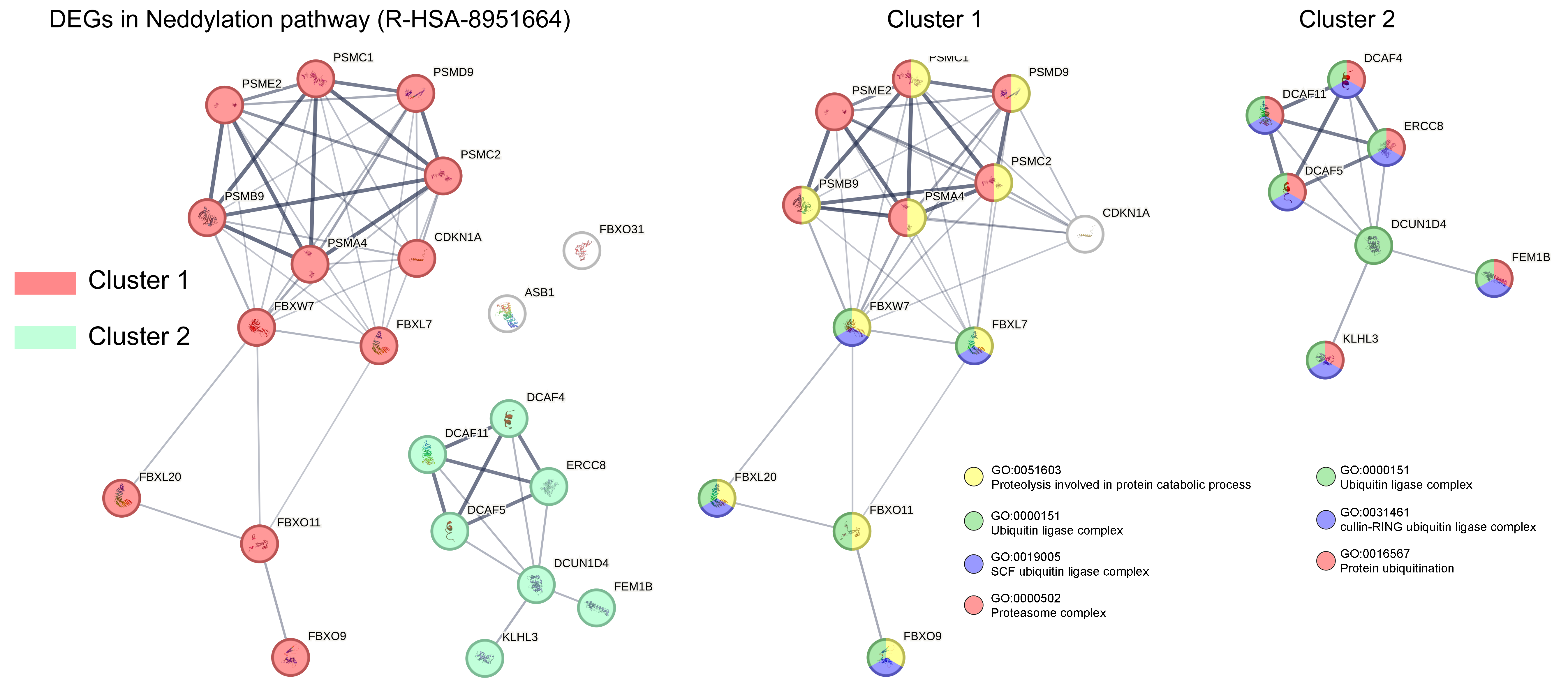

Supplement: S1 Fig — To further understand the cellular components and processes represented by these genes a k-means clustering analysis was performed obtaining two clusters. Gene Ontology (GO) cellular components and processes represented by each of the clusters are detailed on the right side of the image. The network was generated using STRING database (v12.0) and on STRING website. Line thickness between the nodes represents confidence scores of interaction which were calculated using following channels: textmining, experiments, databases, co-expression, neighborhood, gene fusion, co-occurrence. (TIF) [file pone.0325672.s001.tif]

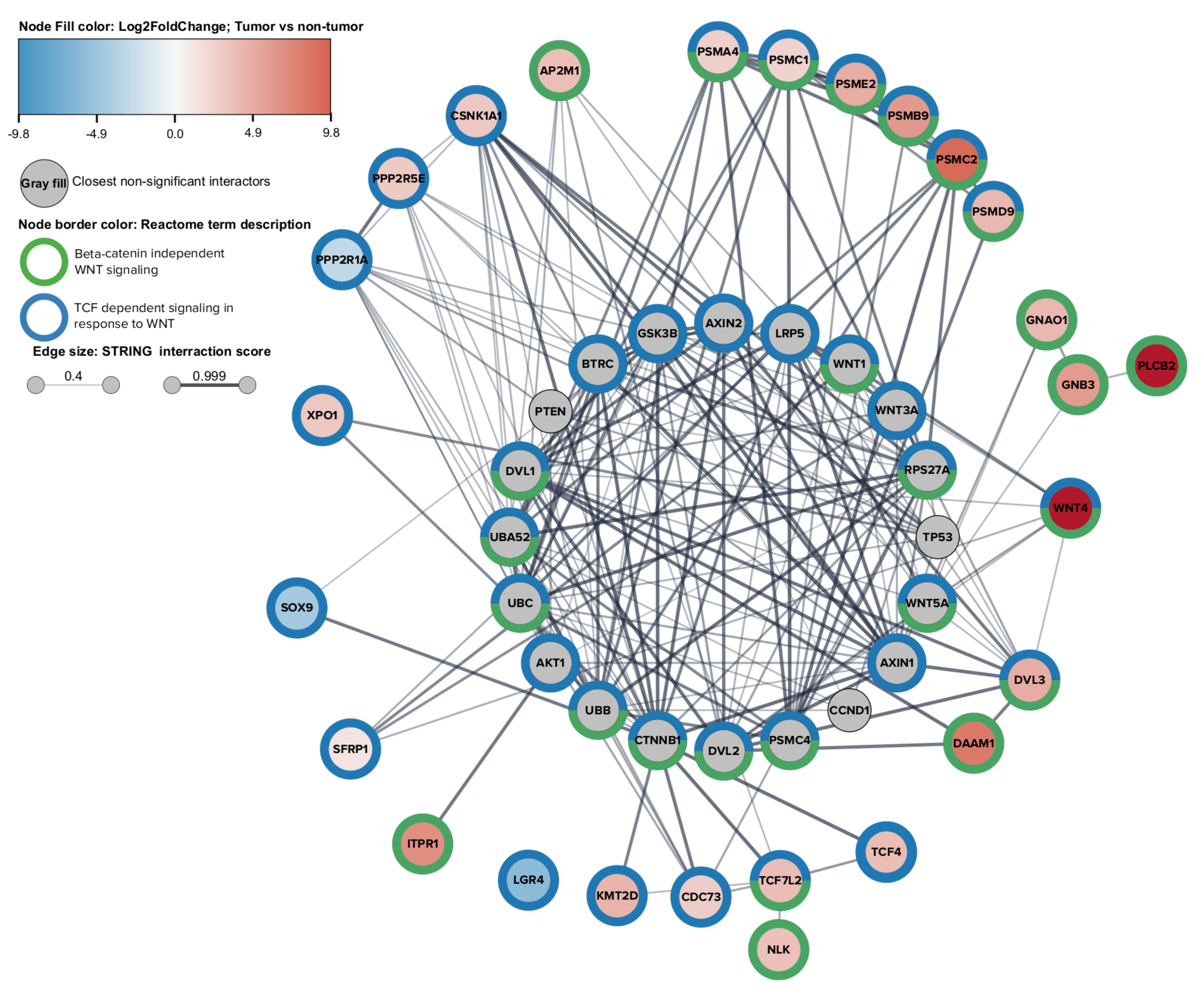

Supplement: S2 Fig — The network also includes 20 closest interacting neighbors (grey nodes) that were not differentially expressed. The graph was made in Cytoscape (v3.10.2) using data from STRING database (v12.0); channels used to calculate interaction confidence scores: textmining, experiments, databases, co-expression, neighborhood, gene fusion, co-occurrence. (TIF) [file pone.0325672.s002.tif]

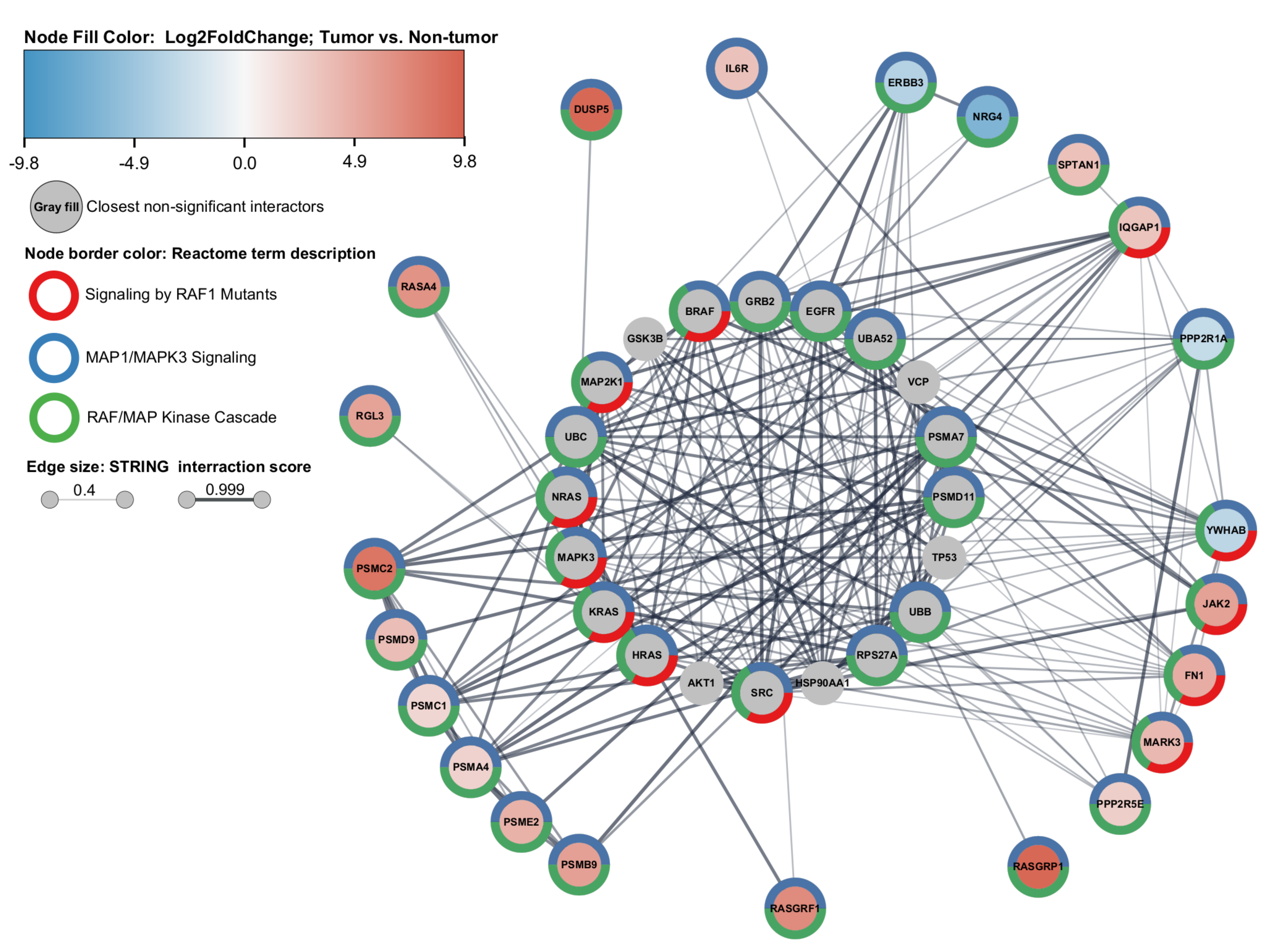

Supplement: S3 Fig — The network also includes 20 closest interacting neighbors (grey nodes) that were not differentially expressed. The graph was made in Cytoscape (v3.10.2) using data from STRING database (v12.0); channels used to calculate interaction confidence scores: textmining, experiments, databases, co-expression, neighborhood, gene fusion, co-occurrence. (TIF) [file pone.0325672.s003.tif]

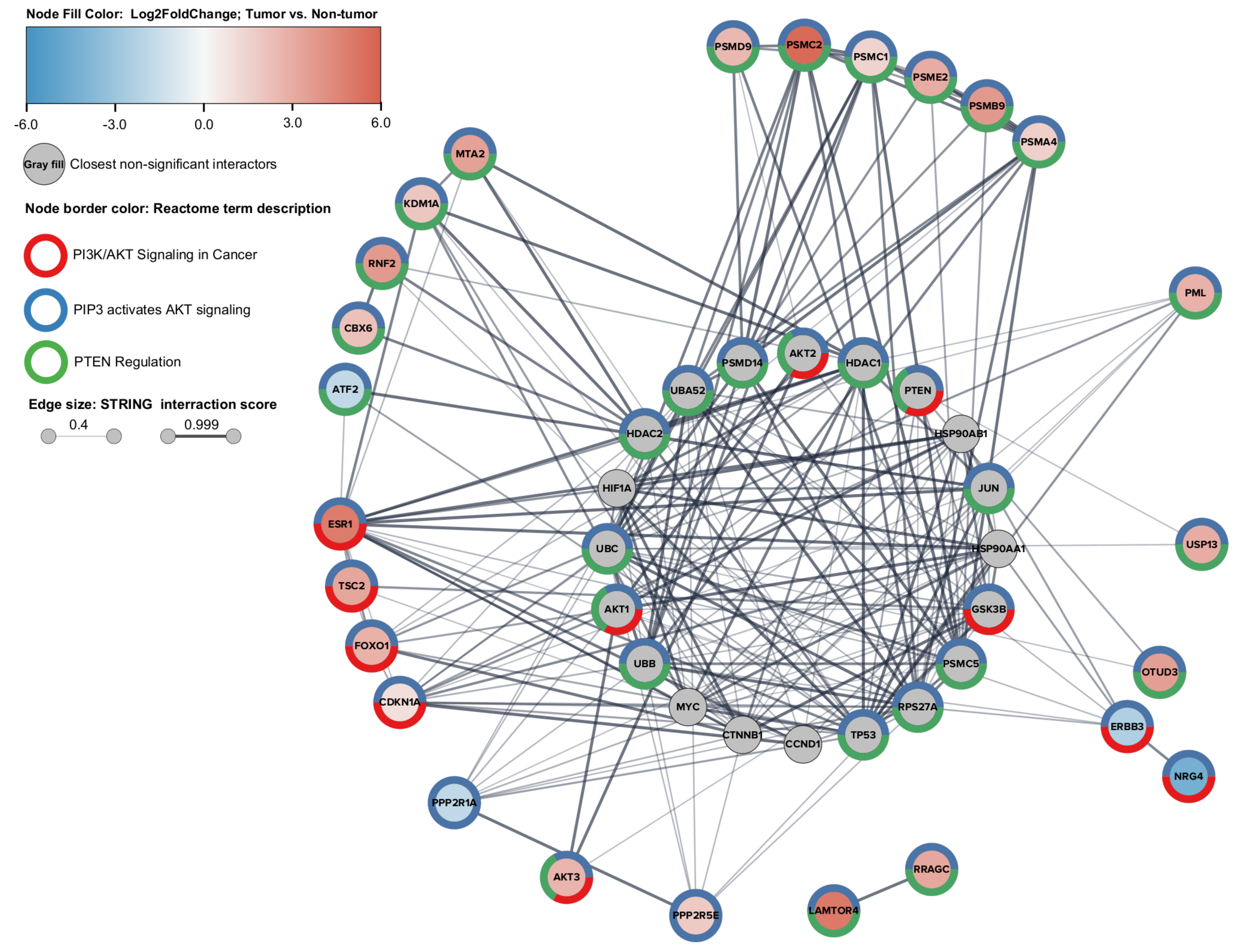

Supplement: S4 Fig — The network also includes 20 closest interacting neighbors (grey nodes) that were not differentially expressed. The graph was made in Cytoscape (v3.10.2) using data from STRING database (v12.0); channels used to calculate interaction confidence scores: textmining, experiments, databases, co-expression, neighborhood, gene fusion, co-occurrence. (TIF) [file pone.0325672.s004.tif]

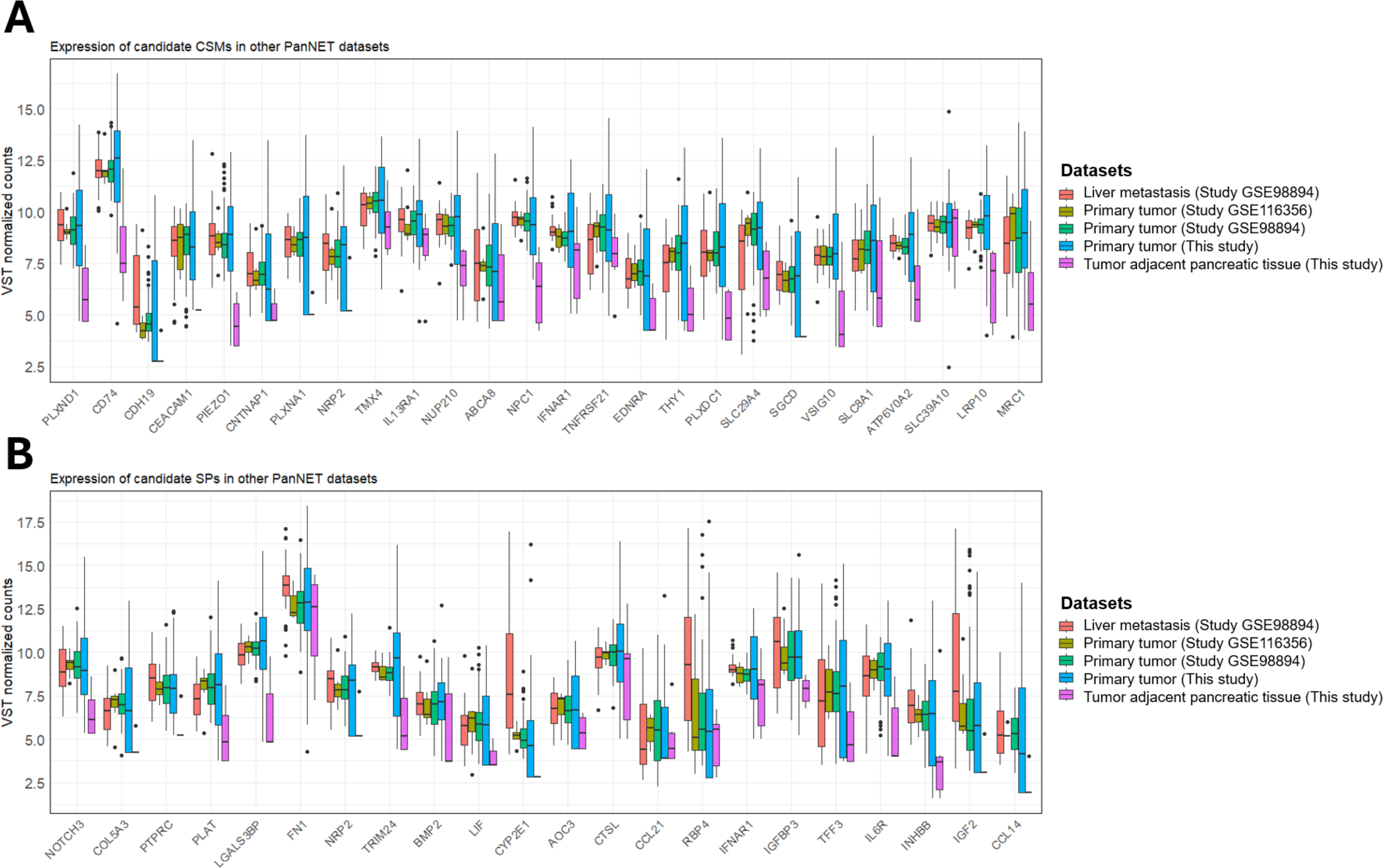

Supplement: S5 Fig — Y-axis – variance stabilizing transformed normalized counts, X-axis – gene symbols. Of the total 27 CSMs NECTIN2 is missing as it was not found in count data of the publicly available datasets. Of the total 24 SPs genes TNC and HLA-A are missing as they were not found in count data of the publicly available datasets. (TIF) [file pone.0325672.s005.tif]
