# Supplementary material for: Transcriptomic profiling of pancreatic neuroendocrine tumors: dysregulation of WNT, MAPK, PI3K, neddylation pathways and potential non-invasive biomarkers
Source: PLoS One. 2025 Jun 16;20(6):e0325672. doi: 10.1371/journal.pone.0325672 (PMC12169574; doi:10.1371/journal.pone.0325672)
Supplement: S1 Text — The ffile includes code and comments regarding differential expression analysis, pathway enrichment analysis, and tissue enrichment analysis. (DOCX) [file pone.0325672.s012.docx]

DESEQ2, Pathway enrichment analysis and Tissue type enrichment analysis analysis - R code

# DEseq2 analysis.

**Description:** the analysis was used to generate differential expression analysis results from salmon count data. The raw transcript count data is avaiable within GEO repository under accession number: GSE281039. The code below was used to generate gene count data using tximeta package, and perform DEseq2 analysis.

# Load in packages

library(tximeta)

library(DESeq2)

library(dplyr)

library(limma)

library(stringr)

library(org.Hs.eg.db)

library(ggplot2)

library(RColorBrewer)

library(pheatmap)

library(IHW)

library(apeglm)

library(ensembldb)

library(AnnotationHub)

library(grid)

library(EnhancedVolcano)

library(cowplot)

library(biomaRt)

library(tidyverse)

# Define workdir

work_dir = setwd("drive:/path/to/directory")

##################PREPROCESSING##################

# Read in metadata table

coldata = read.table("sample_sheet.txt", header = TRUE, fill=TRUE)

# assign factors

coldata$seqbatch = as.factor(coldata$seqbatch)

coldata$grade = as.factor(coldata$grade)

coldata$prepbatch = as.factor(coldata$prepbatch)

coldata$type = as.factor(coldata$type)

coldata$type = as.factor(coldata$functioning)

# add new column containign path to the salmon quant data for each sample

coldata$files = file.path(work_dir, "quants", str_glue("{coldata$names}_quant"),

"quant.sf")

file.exists(coldata$files)

coldata

# Perform transcript quantification using tximeta package

options(timeout = 1000)

se = tximeta(coldata, type = 'salmon')

assayNames(se)

rowRanges(se)

gse = summarizeToGene(se)

gse = addIds(gse, column = 'SYMBOL')

head(gse)

##################RUN DESEQ2 ANALYSIS##################

# Create DEseq2 object

dds = DESeqDataSet(gse, design = ~prepbatch + type)

# Combine replicates (in case some samples contain more than 1 quant file)

dds = collapseReplicates(dds, dds$id, dds$run, renameCols = T)

# Filter genes with low expression counts

keep = rowSums(counts(dds) >= 10) >= 3

dds = dds[keep, ]

# Perform dispersion analysis

detDispType = function (object) {

disp_types = c('parametric', 'local', 'mean')

df = data.frame(matrix(ncol = 3, nrow = 1))

rownames(df)[1] = 'Distances:'

colnames(df) = disp_types

cat('\n')

for (i in disp_types) {

print(paste0('Testing GLM dispersion type: ', i, '.'))

des_ob = DESeq(object, fitType = paste0('', i), quiet = T)

png(paste0('no_cl2_model', i, '.png'), w = 1024, h = 768)

plotDispEsts(des_ob, xlim = c(1, 1e5), ylim = c(1e-2, 100))

dev.off()

mcols = as.data.frame(mcols(des_ob))

rez = median(abs(log(mcols$dispGeneEst)) - abs(log(mcols$dispFit)),

na.rm = TRUE)

df[1, i] = rez

}

rez_col_name = names(df)[which.min(apply(df, MARGIN = 2, min))]

cat('\n')

print(df)

write.table(df,

'Dispersion.txt',

quote = F, sep = '\t')

}

detDispType(dds)

# Perform VST transformation for sample distance and PCA analysis

vsd = vst(dds, blind = FALSE)

assay(vsd)

# Sample distance plot

sampleDists = dist(t(assay(vsd)))

sampleDistMatrix = as.matrix(sampleDists)

rownames(sampleDistMatrix) = paste(vsd$id,

vsd$type,

vsd$prepbatch,

sep = ' - ')

colnames(sampleDistMatrix) = NULL

colors = colorRampPalette(rev(brewer.pal(9, 'Blues')) )(255)

png('filename.png', w = 1920, h = 1080)

pheatmap(sampleDistMatrix,

clustering_distance_rows = sampleDists,

clustering_distance_cols = sampleDists,

col = colors, fontsize = 16)

dev.off()

# PCA PLOTS

# PCA for type and prepbatch factors

png('filename.png', w = 700, h = 780)

pcaData <- plotPCA(vsd, intgroup=c("type", "prepbatch"), returnData=TRUE)

percentVar <- round(100 * attr(pcaData, "percentVar"))

ggplot(pcaData, aes(PC1, PC2, color=prepbatch, shape=type)) +

geom_point(size=3) +

xlab(paste0("PC1: ",percentVar[1],"% variance")) +

ylab(paste0("PC2: ",percentVar[2],"% variance")) +

coord_fixed()+

theme_bw() +

geom_point(size=8)+

theme(legend.text=element_text(size=20), legend.title=element_text(size=23))+

theme(axis.text=element_text(size=20),

axis.title=element_text(size=23))+

scale_colour_discrete(name="Batch")+

scale_shape_discrete(name = "type")

dev.off()

# Differential gene expression analysis PanNETs (Tumor) vs tumor adjacent pancreatic tissues (NT)

dds$type = factor(dds$type, levels = c('NT', 'Tumor'))

ddsMat = DESeq(dds, minReplicatesForReplace = 6,

fitType = 'local')

resultsNames(ddsMat)

p = ncol(attr(ddsMat, "modelMatrix"))

m = ncol(ddsMat)

cooksCutoff = qf(.99, p, m - p)

resultsNames(ddsMat)

res = results(ddsMat, alpha = 0.05, cooksCutoff = cooksCutoff,

pAdjustMethod = 'BH',

name = 'type_Tumor_vs_NT',

filterFun = ihw)

resultsNames(ddsMat)

#Export primary results

res = results(ddsMat, name = "type_Tumor_vs_NT", alpha = 0.05, pAdjustMethod = 'BH')

res

resOrdered = res[order(res$padj), ]

#Apply p-value and Log2FC cutoffs

resOrderedSignif = resOrdered[!is.na(resOrdered$padj) & resOrdered$padj < 0.05, ]

resOrderedSignif2 = resOrderedSignif[abs(resOrderedSignif$log2FoldChange) > 0.5, ]

#Export filtered version

write.table(resOrderedSignif2, 'Tumor_vs_NT_filtered.txt', sep = '\t',

quote = F, row.names = T, col.names = T)

#Perform LFC shrink using apeglm package, to provide more accurate log2FC estimates.

lfc_res <- lfcShrink(ddsMat, res = res, type = "apeglm", coef = "type_Tumor_vs_NT")

lfc_res$SYMBOL = mcols(dds)$SYMBOL

#Export unfiltered version of LFCshrink modified results

write.table(lfc_res, 'Tumor_vs_NT_shrink_unfiltered.txt', sep = '\t',

quote = F, row.names = T, col.names = T)

#Apply Log2FC and P-value cutoffs to LFCshrink modified results and export the final results table

res1Ordered = lfc_res[order(lfc_res$padj), ]

res1OrderedSignif = res1Ordered[!is.na(res1Ordered$padj) & res1Ordered$padj < .05, ]

res1OrderedSignif2 = res1OrderedSignif[abs(res1OrderedSignif$log2FoldChange) > 0.5, ]

res1OrderedSignif2$ENSEMBL = gsub('\\..*', '', rownames(res1OrderedSignif2))

res1OrderedSignif2$Genes = rownames(res1OrderedSignif2)

write.table(res1OrderedSignif2, 'Tumor_vs_NT_shrink_filtered.txt', sep = '\t',

quote = F, col.names = T)

# Create a histogramm of p-values

png('p_value_hist_lfcshrink_para_group2.png', w = 1000, h = 400)

hist(lfc_res$pvalue, col = "lavender",

main = "Distribution of P-values",

xlab = "P-values",

cex.axis = 2,

cex.main = 2)

dev.off()

###########################################################################################

# Heatmap with genes of interest (top 25 upregulated, top 25 downregulated DEGs)

draw_colnames_45 <- function (coln, gaps, ...) {

coord = pheatmap:::find_coordinates(length(coln), gaps)

x = coord$coord - 0.5 * coord$size

res = textGrob(coln, x = x, y = unit(1, "npc") - unit(3,"bigpts"), vjust = 0.5,

hjust = 1, rot = 90, gp = gpar(...))

return(res)

}

assignInNamespace(x = "draw_colnames", value = "draw_colnames_45",

ns = asNamespace("pheatmap"))

head(res1OrderedSignif2$SYMBOL)

heat_colors = brewer.pal(9, "YlOrRd")

#extract top 25 upregulated, downregulated genes

labels_row <- c(res1OrderedSignif2[(res1OrderedSignif2$log2FoldChange > 7.275) | (res1OrderedSignif2$log2FoldChange < -3.61), "SYMBOL"])

labels_row[is.na(labels_row)] <- res1OrderedSignif2$ENSEMBL[is.na(labels_row)]

degs = c(res1OrderedSignif2[(res1OrderedSignif2$log2FoldChange > 7.275) | (res1OrderedSignif2$log2FoldChange < -3.61), "Genes"])

design0 = model.matrix(~dds$type)

mat = assay(vsd)[degs,]

mat = removeBatchEffect(mat, vsd$prepbatch, design = design0)

mat = mat - rowMeans(mat)

anno = as.data.frame(colData(vsd)[, c('grade', 'type')])

colnames(anno)[1] = 'Grade'

colnames(anno)[2] = 'Sample type'

png('heatmapINS_vs_NT_FIN.png',

w = 2000, h = 2000)

heatmap = pheatmap(mat,

annotation_col = anno,

fontsize = 25,

fontsize_row = 25,

border_color = F,

labels_row = labels_row,

color = heat_colors,

annotation_legend = T,

show_rownames = T,

show_colnames = T,

cellwidth = 25,

cellheight = 20,

treeheight_col = 80,

treeheight_row = 80,

clustering_distance_rows = "euclidean",

clustering_distance_cols = "euclidean",

clustering_method = "complete",

legend = T, cex=1)

dev.off()

###########################################################################################

###########################################################################################

#Volcano plot

res$SYMBOL = mcols(dds)$SYMBOL

# VOLCANO PLOTs

png('volcano_plot2.png',

w = 1000, h = 1080)

#Cap the p-value axis in case

lfc_res$pvalcapped <- ifelse(lfc_res$pvalue < 1.01E-15, 1.01E-15, lfc_res$pvalue)

lfc_res = lfc_res[order(lfc_res$padj), ]

#vector of genes to be labeled

labelsvolcano <- c("goi1", "goi2", "goi3")

EnhancedVolcano(lfc_res,

lab = lfc_res$SYMBOL,

x = 'log2FoldChange',

y = 'pvalcapped',

selectLab = labelsvolcano,

drawConnectors = TRUE,

widthConnectors = 0.75,

FCcutoff = 0.5,

pCutoff = 0.05,

xlim = c(min(lfc_res[["log2FoldChange"]], na.rm = TRUE) + 0,

max(lfc_res[["log2FoldChange"]], na.rm = TRUE) - 0),

ylim = c(0, 15),

pointSize = 7,

labSize = 7,

caption = "",

title = "",

subtitle = "",

gridlines.major = FALSE,

gridlines.minor = FALSE,

legendLabSize = 30,

legendIconSize = 15,

axisLabSize = 40,

hline = c(0.0000708443611928486),

col = c("grey20", "darkgreen", "royalblue3", "red4"))

dev.off()

###########################################################################################

###########Export batch effect corrected vst counts for further visualizations and pathfindR analysis#############

vsd = vst(dds, blind = FALSE)

vsd

design0 = model.matrix(~dds$type)

mat = assay(vsd)

mat

#Remove batch effect (introduced sample preparation batch) by using limma package

mat = removeBatchEffect(mat, vsd$prepbatch, design = design0)

mat

rownames(mat) = gsub('\\..*', '', rownames(mat))

rownames(mat) = mcols(dds)$SYMBOL

mat

write.table(mat, 'counts_vst_norm.tsv', sep = '\t',

quote = F, row.names = T, col.names = T)

# Active subnetwork oriented pathway enrichment analysis using PathfindR package

**Description:** The code below was used to generate pathway enrichment analysis results from differentially expressed genes in tumor vs. non-tumor comparison.

*#Loading packages*

**library**(pathfindR)

**library**(stringr)

**library**(ggplot2)

**library**(ggrepel)

**library**(plotfunctions)

**library**(dplyr)

**library**(reshape2)

**library**(RColorBrewer)

**library**(patchwork)

*#SetWD*

setwd("drive:/path/to/work/directory")

*#Importing dataframe with DEGs, the table must contain columns in following order: HGNC gene symbols, Log2FC values, P-value(FDR, BH p-value from DGE test).*

*#In this analysis we used all genes found in Supplementary Table 2*

df <- read.table(file= "PathfindR_input.txt", header = TRUE)

df <- na.omit(df) *#Omit rows with NA for symbols (genes with ENSG identifier but no HGNC identifiers)*

*#Process input data*

example_processed <- input_processing(

input = df, *# the input data from DGE analysis*

pin_name_path = "STRING", *# database for PIN Generation and active subnetwork search*

convert2alias = F *# boolean indicating whether or not to convert missing symbols to alias symbols in the PIN. F by default as introducing aliases may produce biased results.*

)

write.table(example_processed,"InputGenes_processed.txt", col.names = TRUE, row.names = FALSE, sep="\t") *# export table of processed input genes to check whether the HGNC symbols match.*

*#Fetch gene sets for enrichment anlaysis*

reactome_list <- fetch_gene_set(

gene_sets = "Reactome", *#The study used Reactome database; other options are KEGG, BioCarta, etc.*

min_gset_size = 10,

max_gset_size = 300

)

reactome_gsets <- reactome_list[[1]]

reactome_descriptions <- reactome_list[[2]]

*#Active Subnetwork Search and Enrichment Analyses*

n_iter <- 10 *## number of iterations, the study used 10 as its the default option.*

combined_res <- NULL

**for** (i **in** 1:n_iter) {

*###### Active Subnetwork Search*

snws_file <- paste0("active_snws_", i) *# Name of output file*

active_snws <- active_snw_search(

input_for_search = example_processed,

pin_name_path = "STRING",

snws_file = snws_file,

score_quan_thr = 0.8, *# you may tweak these arguments for optimal filtering of subnetworks, the default as indicated by package developers was 0.8*

sig_gene_thr = 0.02, *# you may tweak these arguments for optimal filtering of subnetworks, the default as indicated by package developers was 0.02*

search_method = "GR", *# The developers suggest ussing "GR" (Greedy search)*

seedForRandom = i *# setting seed to ensure reproducibility per iteration*

)

*###### Enrichment Analyses*

current_res <- enrichment_analyses(

snws = active_snws,

sig_genes_vec = example_processed$GENE,

pin_name_path = "STRING",

genes_by_term = reactome_gsets,

term_descriptions = reactome_descriptions,

adj_method = "bonferroni",

enrichment_threshold = 0.05,

list_active_snw_genes = TRUE

) *# listing the non-input active snw genes in output*

*###### Combine results via `rbind`*

combined_res <- rbind(combined_res, current_res)

}

*#Export active SNVs*

melt2 <- melt(active_snws)

write.table(melt2,"filename.txt", sep=";",col.names = FALSE, row.names = FALSE)

*#Summarize and export enrichment results*

summarized_df <- summarize_enrichment_results(combined_res,

list_active_snw_genes = TRUE)

write.table(summarized_df,"filename.txt", col.names = TRUE, row.names = FALSE, sep="\t")

*#Export expanded enrichment results (includes list of up- and down-regulated genes)*

final_res <- annotate_term_genes(

result_df = summarized_df,

input_processed = example_processed,

genes_by_term = reactome_gsets

)

write.table(final_res,"filename.txt", col.names = TRUE, row.names = FALSE, sep="\t")

*###################Visualizations#######################*

*#Data preparation for enrichment charts (enrichment dot plots)*

result_df_broken <- final_res

result_df_broken$Term_Description <- str_wrap(summarized_df$Term_Description, width = 30)

enrichment_chart(result_df_broken, top_terms = 15)

*# Perfrom clustering analysis to identify biological similar terms (the similarity depends on the amount of overalapping genes)*

example_pathfindR_output_clustered <- cluster_enriched_terms(final_res, plot_dend = F, plot_clusters_graph = T)

example_pathfindR_output_clustered

edit(example_pathfindR_output_clustered) *#edit is used to shorten some term desriptions that are too long..*

selected_clusters <- subset(example_pathfindR_output_clustered, Cluster %**in**% c(1, 19, 16, 8, 11)) *#selecting clusters of interest (top 5 largest clusters in this case)*

*#selected_clusters <- selected_clusters2[selected_clusters2$Status == "Representative", ]*

selected_clusters$Term_Description <- str_wrap(selected_clusters$Term_Description, width = 30)

selected_clusters$Term_Description <- paste(selected_clusters$ID, selected_clusters$Term_Description)

selected_clusters <- edit(selected_clusters)

*#Enrichment chart producing stastistics of top 5 terms (according to FDR value) in each of the selected clusters (in this case 1, 19, 16, 8, 11 clusters)*

*#Representative figure in article (Figure 3B)*

plotTERMS<-enrichment_chart(selected_clusters, plot_by_cluster = T, top_terms = 5) +

scale_color_gradient(low = "#3056f1", high = "#f31b19")+

theme(axis.text.y = element_text(size = 10, colour = "#1f1f1f"))

*#export enrichment table with clustering results*

write.table(example_pathfindR_output_clustered, file = "filename.txt", col.names = TRUE, sep="\t")

*#Heatmap of terms and genes showing Log2FC expression values for genes related to selected pathways*

*#Selecting terms of interes (in this case the top 5 terms from clusters 1, 8, 11, 16, 19)*

terms_of_interest <- c("R-HSA-8951664", "R-HSA-201681", "R-HSA-195721", "R-HSA-5688426", "R-HSA-5684996", "R-HSA-9664323", "R-HSA-1489509", "R-HSA-112043", "R-HSA-112040", "R-HSA-163615", "R-HSA-9006927", "R-HSA-8848021", "R-HSA-1236394", "R-HSA-8847993", "R-HSA-6785631", "R-HSA-9656223", "R-HSA-6802955", "R-HSA-6802949", "R-HSA-6802946", "R-HSA-9649948", "R-HSA-512988", "R-HSA-451927", "R-HSA-1433557", "R-HSA-9669938", "R-HSA-9670439")

*#Select clusters (1, 8, 11, 16, 19 in this case)*

selected_clusters2 <- subset(example_pathfindR_output_clustered)

filtered_df <- selected_clusters2 %>% filter(ID %**in**% terms_of_interest)

filtered_df$Cluster <- as.factor(filtered_df$Cluster)

filtered_df <- filtered_df[order(filtered_df$Cluster, decreasing = F),]

filtered_df$Term_Description <- str_wrap(filtered_df$Term_Description, width = 40)

*#Remove repeating gene symbol column from processed input gene column. The visualization below requires Log2FC values for each of the genes*

example_processed2<-example_processed[2:4]

*#Plot term-gene heatpmap*

*#Representative figure in article (Figure 3D)*

plot1<-term_gene_heatmap(result_df = filtered_df, genes_df = example_processed2,

low = "#0d00ff",

mid = "#fdead9",

high = "#CE4938",

num_terms = NULL,

use_description = F,

legend_title = "Log2FC\nTumor vs.\nNon-tumor",

sort_terms_by = T,

pin_name_path = "STRING",

)+

theme(axis.text.y = element_text(size = 9, colour = "#1f1f1f"),

axis.text.x = element_text(size = 10))+

theme(legend.key.size = unit(0.1, 'cm'), *#change legend key size*

legend.key.height = unit(0.3, 'cm'), *#change legend key height*

legend.key.width = unit(0.3, 'cm'), *#change legend key width*

legend.title = element_text(size=8), *#change legend title font size*

legend.text = element_text(size=8))

*#Scores matrix and heatmap*

*#define cases and controls from the experiment*

cases <- c("NET1", "NET10", "NET11", "NET13", "NET14", "NET17", "NET19", "NET2", "NET20", "NET21", "NET22", "NET23", "NET24", "NET25", "NET26", "NET7", "NET12", "NET15", "NET16", "NET18", "NET27", "NET3", "NET4", "NET5", "NET6", "NET8", "NET9", "NET30","NET29", "NET28")

cases*# vector of cases (tumors)*

controls = c("X2NT", "X3NT", "X4NT", "X5NT", "X6NT", "X7NT")

controls*# vector of contros (tumor adjacent tissues)*

*#assign counts matrix (vst transformed, normalized countrs matrix from DEseq2)*

example_experiment_matrix<-read.table(file ="counts_vst_norm_TumorvsNT.tsv", header = TRUE, fill=TRUE, sep = "\t", row.names = 1)

example_experiment_matrix <- as.matrix(example_experiment_matrix)

filtered_df2 <- filtered_df

filtered_df2$Term_Description <- str_wrap(filtered_df2$Term_Description, width = 30)

*#Create scores matrix and plot results displaying the "upregulation" or "downregulation" of a specific pathway in each sample based on input count data*

*#Representative figure in article (Figure 3C)*

score_matrix <- score_terms(

enrichment_table = filtered_df2,

exp_mat = example_experiment_matrix,

cases = cases,

use_description = F, *# default FALSE*

label_samples = TRUE, *# default = TRUE*

case_title = "PanNETs", *# default = "Case"*

control_title = "Adjacent NTTs", *# default = "Control"*

low = "#7891f7", *# default = "green"*

mid = "#fdead9", *# default = "black"*

high = "#f66064",

plot_hmap = F)

score_matrix

plot2<-plot_scores(

score_matrix,

cases = cases,

label_samples = TRUE,

case_title = "PanNETs",

control_title = "NT",

low = "#7891f7",

mid = "#fdead9",

high = "#f66064"

) +

ggplot2::theme(axis.text.y = element_text(size = 10, colour = "#1f1f1f"),

axis.text.x = element_text(size = 10))

# Tissue enrichment analsysis with TissueEnrich package

**Description:** The code below was used to generate tissue enrichment analysis results from differentially expressed genes marked as secretome or cell surface markers from tumor vs. non-tumor comparison.

*#Load in packages*

**library**(ggplot2)

**library**(readxl)

**library**(TissueEnrich)

**library**(GSEABase)

**library**(tidyr)

*# Define work dir*

work_dir = 'drive:/path/to/directory/'

setwd(work_dir)

*# Read in data*

src = read_xlsx('Sup. table 6.xlsx', sheet = 1, col_names = T) *#the data listed in supplementary table 6 were used for tissue enrichment analysis*

names(src)[3] = 'ENSEMBL'

src = src[!is.na(src$ENSEMBL),]

src

in_data = read_xlsx('resources/Additional_file_1.xlsx',

sheet = 3, col_names = T)

names(in_data)[1] = 'ENSEMBL'

in_data

*#Tissue enrichment*

sheet_csm_genes = unique(c(src$ENSEMBL))

sheet_csm_gs = GeneSet(geneIds = sheet_csm_genes, organism = 'Homo Sapiens',

geneIdType = ENSEMBLIdentifier())

sheet_csm_gs_out = teEnrichment(inputGenes = sheet_csm_gs)

seEnrichmentOutput = sheet_csm_gs_out[[1]]

enrichmentOutput = setNames(data.frame(assay(seEnrichmentOutput),

row.names = rowData(seEnrichmentOutput)[,1]),

colData(seEnrichmentOutput)[,1])

enrichmentOutput$Tissue = row.names(enrichmentOutput)

enrichmentOutput$p_adj_raw = 10 ^ -(enrichmentOutput$Log10PValue)

enrichmentOutput = enrichmentOutput[order(enrichmentOutput$fold.change, decreasing = T),]

enrichmentOutput

write.table(enrichmentOutput,'SRC_DEGs_enriched_tissue_raw.txt',

row.names = F, col.names = T, sep = '\t', quote = F)

*#Heatmap (Figure 5D)*

enrichmentOutput_sig = enrichmentOutput[enrichmentOutput$fold.change != 0,]

enrichmentOutput_sig = enrichmentOutput_sig[enrichmentOutput_sig$p_adj_raw < 0.05,]

seExp = sheet_csm_gs_out[[2]][["Pancreas"]]

exp = setNames(data.frame(assay(seExp), row.names = rowData(seExp)[,1]), colData(seExp)[,1])

exp$Gene = row.names(exp)

exp = exp %>% gather(key = "Tissue", value = "expression", 1:(ncol(exp) - 1))

de_gene_df = in_data[, c(1, 7), drop = F]

names(de_gene_df)[1] = 'Gene'

names(de_gene_df)[2] = 'Symbol'

exp = merge(exp, de_gene_df, by = 'Gene')

pancreas_heat = ggplot(exp, aes(Tissue, Symbol)) +

geom_tile(aes(fill = expression), color = 'white') +

scale_fill_gradient(low = 'white', high = 'red') +

labs(x = '', y = '') +

theme_bw(base_size = 26) +

guides(fill = guide_legend(title = "Log2(TPM)")) +

theme(plot.title = element_text(hjust = 0.5, size = 20),

axis.text.x = element_text(angle = 45, vjust = 1, hjust = 1, size = 14),

panel.grid.major = element_blank(),

panel.grid.minor = element_blank(),

axis.text.y = element_text(size = 14),

legend.title=element_text(size=14),

legend.text=element_text(size=14))

pancreas_heat
