# Supplementary material for: Transcriptomic profiling of pancreatic neuroendocrine tumors: dysregulation of WNT, MAPK, PI3K, neddylation pathways and potential non-invasive biomarkers
Source: PLoS One. 2025 Jun 16;20(6):e0325672. doi: 10.1371/journal.pone.0325672 (PMC12169574; doi:10.1371/journal.pone.0325672)
Supplement: Attachment — Submitted filename: Response to reviewers.docx [file pone.0325672.s014.docx]

| **Reviewer 1** | |
| --- | --- |
| **Comment** | **Response** |
| Include the total number of differentially expressed genes (DEGs) and specify the significance thresholds used for their identification to strengthen the abstract. | Thank you for suggesting to add statistical thresholds. We updated the abstract. Please see added lines (27 - 29) in tracked changes. |
| Line 20: Provide updated statistics on cancer in general as well as this cancer type prevalence, including survival rates, to highlight the critical need for prognostic biomarkers. Cite “Cancer statistics, 2024, 2024”. Then give intro in cancer therapy in general, cite NIH paper “Cancer treatments: Past, present, and future, 2024” (PMID: 38909530) for more information. | Thank you for the comment within "Introduction" section line 51, we have briefly stated the incidence of PaNETs and treatment information. According to your suggestion we have expanded this in line (lines 53-54 in tracked changes). We added more details regarding overall survival statistics of patients with PanNETs. Regarding article PMID:38909530. It is a valuable review, however, it includes no information regarding neuroendocrine tumors which are rather different from "classical" cancers in terms of differentiation and clinical aggressiveness. Regardless, we still included the article within the literature review as it discusses that surgery remains as the primary treatment option for most forms of cancer. This is also the same for neuroendocrine tumors. Regarding the treatment of PanNETs, we have already briefly discussed this within lines 51 - 61. |
| Line 40: Expand on the clinical and biological challenges posed by PanNETs, such as their asymptomatic nature and high metastatic potential. | Thank you for the comment. The metastatic risk is discussed in lines 52 - 53 where we found in a review by Das S and Dasari A that metastatic involvement is present in between of 21 and 65% of diagnosed cases. We've briefly discussed challenges regarding current serum biomarkers for diagnostics (chromogranin A, neuron-specific enolase, and pancreatic peptide) in lines 62-63. Lastly, we've also highlighted their asymptomatic nature which mainly concerns non-functioning tumors, please see lines 65 - 67. |
| Line 65: Include a discussion on the potential implications of transcriptomic profiling for personalized medicine in PanNETs. | We have discussed impact of gene expression studies in advancement of PanNETs in lines 76 - 79 |
| Line 75: Cite recent studies addressing molecular markers and pathways in PanNETs, emphasizing areas of agreement or contradiction with the current study. | Thank you for the comment. We have discussed this in lines 68 - 79, highlighting, the currently most significant molecular markers ATRX/DAXX and PTEN, TSC2, PIK3CA, and MEN1. |
| Line 150: Provide additional details on the preprocessing of RNA-seq data, including quality control metrics and read trimming thresholds. | We have provided the details regarding quality metrics in Materials and methods, "data analysis" subsection (lines 130 - 133). |
| Line 170: Discuss the justification for using the STRING and Reactome databases in the pathway enrichment analyses. | Thank you for the suggestion. We chose the STRING database as it is compatible with PathfindR active subnetwork-based enrichment analysis. Accordingly, STRING database was used to generate the PPI interaction network which was segregated into active subnetworks by PathfindR tool. After this the genes within identified subnetworks were used as input to perform pathway enrichment analysis using Reactome database. We chose specifically this database as according to methodological study by Gable et al. (PMID: 36088548) it provided the highest overall performance of manually curated annotation systems for human genome related studies. Accordingly, we included this information manuscript. Please see tracked changes in lines 148 - 152. |
| Line 200: Highlight the biological relevance of the identified overrepresented pathways, such as their roles in PanNET tumorigenesis and progression. | Thank you for the comment. We have updated results section highlighting the biological relevance of identified overrepresented pathways. This was done by moving some parts of the discussion to results section as such change was also requested by another reviewer. Please see tracked changes in lines 234 - 237, 240 - 252, and 266 - 275. |
| Line 240: Include more granular details on the differentially expressed genes (DEGs) across tumor grades, particularly focusing on unique markers for G3 tumors. | Thank you for the comment In section "Transcriptomic changes across different grades of PanNETs" we have added some details regarding NCAM1 gene expression in G3 tumors compared to G2 and G1 tumors. Please see tracked changes in lines 289 - 293. NCAM1 is known to be another marker of Neuroendocrine differentiation in NETs (particularly lung NETs) and we can observe that the expression of NCAM1 is significantly downregulated in G3 tumors compared to G2 and G1. This is indicates that G3 tumors, while still histologically showing features of well-differentiated tumors, are slightly less differentiated than G2 and G1 NETs in terms of NCAM1 expression. |
| Line 280:Discuss why certain genes (e.g., IGF2, IGFBP3) were chosen as potential biomarkers and their implications for liquid biopsy development. Mention previous study reporting IGFBP as glioma biomarkers: “A bioinformatic study of IGFBPs in glioma regarding their diagnostic, prognostic, and therapeutic prediction value, 2023” | Thank you for the comment. We've discussed to some extent why we focused on specifically IGF2 and IGFBPs in lines 388 - 399. However, it is interesting to see that the involvement of IGFBPs have been also assessed in gliomas with interesting results. This further provides another layer of evidence that IGF axis does play a significant role in carcinogenesis. Accordingly, we've included article “A bioinformatic study of IGFBPs in glioma regarding their diagnostic, prognostic, and therapeutic prediction value, 2023” within our manuscript. Please see tracked changes in lines 438 - 440. |
| Line 350: Provide a critical comparison of the findings with prior studies on PanNET-related pathways, particularly regarding WNT and MAPK signaling. Provide a more detailed context for the relevance of the identified pathways (e.g., WNT, MAPK, PI3K) in tumorigenesis. Mention recent studies reporting association of these pathway in cancer. Such as“Isocucurbitacin B inhibits glioma growth through PI3K/AKT pathways and increases glioma sensitivity to TMZ by inhibiting hsa-mir-1286a, 2024” “MAPK signaling pathway-based glioma subtypes, machine-learning risk model, and key hub proteins identification, 2023,PI3K/AKT/mTOR signaling in gastric cancer: Epigenetics and beyond, 2020” | Thank you for the comment. We have provided some comparison of our results regarding WNT and MAPK signaling in PanNETs compared to other studies. Please see lines 241-252 and 366 - 370 (wnt); lines 248 - 252 and 379- 387 (MAPK signaling); lines 266 - 275 and 388 - 399 (PI3K and mTOR signaling). The studies that are provided within the comment are related to gliomas and gastric cancer which are entire different tumors compared to pancreatic neuroendocrine tumors. While we would like to primarily focus on PanNET studies for comparing our results to literature. But we chose to include the studies: “MAPK signaling pathway-based glioma subtypes, machine-learning risk model, and key hub proteins identification, 2023" and "PI3K/AKT/mTOR signaling in gastric cancer: Epigenetics and beyond, 2020” within introductory section of the discussion to generally excel the fact that these are oncogenic pathways that are dysregulated in many types of cancers. Changes were made in line 361. |
| Line 400: Address potential limitations of the study, such as the low sample size for G3 tumors, and propose strategies for addressing these in future research. Discuss the bias from transcriptional data, refer to “Genetic expression in cancer research: Challenges and complexity, 2024” and “Technical and Biological Biases in Bulk Transcriptomic Data Mining for Cancer Research, 2025” | Thank you for the comment. We have already discussed the low sample size of G3 tumors as one of the primary limitations (Please see lines 450-462). The article "Genetic expression in cancer research: Challenges and complexity, 2024" is indeed interesting as it discusses common pitfalls of bulk RNA-seq studies. We cited this study when discussing the limitations of using non-dissected adjacent pancreatic tissue. This type of tissue contains a mix of both exocrine and endocrine pancreas cells and due to cell type mixing not all DEGs represent changes in tumor compared to cells of origin (endocrine pancreas). Some of the DEGs are related to digestive functions of exocrine pancreas, such as CPA genes which are downregulated in our tumor samples. |
| Line 450: Suggest how the identified biomarkers could be integrated into clinical workflows for PanNET diagnosis and management. Suggest future studies that could validate these findings in patient-derived xenograft models or larger cohorts. Previous studies using xenograft models of cancer should be mentioned, such as “Comparing volatile and intravenous anesthetics in a mouse model of breast cancer metastasis, 2018” | Thank you for the comment. In context of PanNETs we've raised these suggestions in discussion section: lines 388 - 399 and 433 -449. However, we do not think it would be appropriate to discuss the article "Comparing volatile and intravenous anesthetics in a mouse model of breast cancer metastasis, 2018" as it is a conference abstract and it is out context for this study. |
| Annotate the heatmaps with additional labels or legends to clarify the significance of the identified DEGs. | Thank you for the comment. The heatmaps in Figures 2 and 4 represent top 25 upregulated and downregulated genes with FDR value < 0.05. In Figure 2 D heatmap we've added 2 annotations: 1) sample type which describes whether the sample is tumor tissue or tumor adjacent pancreatic tissue (TAPT); 2) tumor grade which describes what is the histological grade off the tumor sample (G1 - grade 1, G2 - grade 3, G3 - grade 3). |
| Include additional context for the significance of cell surface markers (CSMs) and secretome proteins (SPs) in the clinical management of PanNETs. | We have provided these details in discussion section. Please see lines 425 - 432 and 433 - 449 |

| **Reviewer 2** | |
| --- | --- |
| **Comment** | **Response** |
| 1. The title appears somewhat complex; please rephrase the title while retaining the focus on pathways and biomarkers. | Thank you for the suggestion. We made some minor changes to our title to make it less complex while retaining the focus on pathways and biomarkers. This would be our new title "Transcriptomic profiling of pancreatic neuroendocrine tumors: dysregulation of WNT, MAPK, PI3K, neddylation pathways and potential non-invasive biomarkers" |
| 2. There seems to be an imbalance in the number of samples between PanNETs and TAPTs. Do you think it may affect the statistical tests? Please justify it. | The difference in number of tumor samples and tumor adjacent pancreatic tissues (TAPTs) was primarily due to tissue availability as most of the resected tumor specimens did not contain enough non-tumor tissue. For future studies this could be alleviated by using microdissection techniques to acquire enough tumor free material. Unfortunately, six TAPT samples was all we could collect for this study. This, of course, may impact the overall statistical power of this study. To somewhat alleviate this issue we employed stringent filtering of differential expression analysis results performing Log2 Fold Change value shrinkage using "apeglm" method developed by Anqi Zhu et al. (PMID: 30395178). By applying this method, we filtered out genes that came up as differentially expressed because one of the control samples had abnormally high or low read counts of that specific gene resulting in overestimated fold changes. |
| 3. Could you elaborate on the statistical methodologies used for clustering analysis? Specifically, how were significant clusters and pathways determined? | Thank you for the comment. To carry out pathway enrichment analysis we used PathfindR package (v2.4.1) developed by Uglen et. al (Published paper PMID: 31608109). Here the enrichment analysis is based on active protein-protein interaction subnetworks. Accordingly, this method first generates a protein-protein interaction network using various databases, in our case we resorted to STRING database. Following this, the algorithm then searches for active subnetworks. The package itself provides provides three algorithms for subnetwork search Greedy, Simulated annealing, and Genetic algorithm. In our case we resorted to Greedy algorithm as it was the default one and recommended by the package authors. In our case the subnetwork search was repeated 10 times. We've updated this part in methods section (lines 146 - 157). Following this, the genes from active subnetworks are then used to perform pathway enrichment analysis; here the initial protein-protein interaction network is also used to derive background genes for the analysis. The enrichment analysis was done using Reactome database. We also updated methods section describing the choice behind focusing on reactome instead of KEGG, wikipathways or other databases. Please see tracked changes in lines 149 - 152. We also added information stating that only the pathways with false discovery rate adjusted p-values lower than 0.05 were considered as significant. Please see tracked changes in lines 154 - 155. This package also provides the tools to cluster the enriched terms and identify representative terms in each cluster, to score the enriched terms per sample and to visualize analysis results. Regarding the clustering analysis, we performed it using hierarchical clustering method. This was done to cluster the terms according to their biological similarity. We updated this information in methods section, please see tracked changes in lines 155 - 157. |

| **Reviewer 3** | |
| --- | --- |
| **Comment** | **Response** |
| 1. The first major issue with the study is novelty. Similar studies have been performed earlier. Authors should describe a strong rationale for this study. | Thank you for the comment. We understand that there are previous studies that have employed RNA-sequencing to describe gene expression alterations in PanNETs. However, one of the major drawbacks of previous studies is the lack of non-tumor controls. Using this approach we have identified specific pathways that are dysregulated within PanNETs compared to non-tumor pancreatic tissues. One of the most notable findings here is the upregulation of neddylation pathway which has only recently been proposed to serve a significant role in tumor development (PMID: 38575611). Furthermore, the clinical diagnostics of neuroendocrine tumors are in dire need of novel minimally invasive biomarkers. Accordingly, in our data we have identified specific genes that encode secretome proteins and cell surface markers that are upregulated in tumor tissues compared to non-tumor pancreas. These highlighted markers could serve as potential biomarkers for blood-based marker assay development to assist with the diagnostics and prognostics off PanNETs. Therefore we consider that the publishing of this data and observations would only strengthen the research field of pancreatic neuroendocrine tumors. |
| 2. Another issue with the manuscript is lack of details for each figure panel. For example, in Fig. 2, authors write 1 line for all the panels in this figure and mention that a number of genes were differently expressed. Without calling out each panel in the text, it will be difficult to follow the manuscript. | Thank you for the comment. We apologize for this issue. We updated the text in results section, subsection "Transcriptomic changes in pancreatic neuroendocrine tumor tissues" by adding text describing observations in each panel of Figure 2 separately. Please see tracked changes in lines 180 - 183 and 187 - 190. |
| 3. The authors have discussed the results in detail and tried to make sense of existing data. Some of this information should be incorporated into results section as it will make better sense there. | Thank you for the comment. We've updated the results subsection "Overrepresented pathways in the list of genes differentially regulated within PanNETs" by moving some parts of discussion within this subsection. Please see tracked changes in lines 235 - 238, 241 - 252, and 269 - 275. |
| 4. Authors mention that grade 3 (G3) tumor is more proliferative in comparison to other lower grades, however, they don’t find genes linked proliferation upregulated in G3 tumor samples. Could this be due to miss-classification. G3 and G2 look transcriptionally very similar. | Thank you for the comment. The tumors were classified using WHO classification system which is based on Ki-67 index. The classification itself was done by pathologists at the hospitals where the patients were admitted to. The discrepancy in results regarding comparison of G3 vs. G1 and G3 vs. G2 tumors could be attributed to the low number of G3 tumor cases. As G3 tumors are the rarest form of PanNETs the biological material representing G3 tumor is hard to acquire. As the sequenced tissue samples were FFPE there is also RNA degradation present in all sequenced samples. This unfortunately results in inability to capture lowly expressed transcripts and as a result we are limited to identifying changes in highly expressed transcripts. Nevertheless, one of the interesting changes we can observe is that G3 tumors express NCAM1 at significantly lower level. NCAM1 is considered to be yet another marker of neuroendocrine differentiation in neuroendocrine tumors. This shows that G3 tumors, while histologically still exhibiting features of well-differentiated tumors, are actually less differentiated than G2 or G1 tumors in terms of NCAM1 expression. We've updated the text describing these observations in detail. Please see tracked changes in lines 289 - 293. |
| 5. Authors have not performed any experiment to validate their findings, this makes it underwhelming. | Thank you for commenting on this issue. We acknowledge that this study is purely an observational study with no data from in-vitro experiments. We initially also wanted to carry out testing of proposed candidate secretome proteins in plasma of PanNET patients to see if the proposed secretome proteins could be used for further testing as non-invasive blood-based biomarkers. Unfortunately, we are limited by the availability of biological material as we do not have available blood samples that would represent blood plasma before PanNET surgery. To further validate that the proposed candidate cell surface markers and secretome proteins are not unique only to our dataset we examined the expression of these candidates in two other PanNET datasets from gene expression omnibus (GSE98894 and GSE116356). We included the results of this comparison in results section (please see lines 339-343), and supplementary figure 3. Here we can observe that these candidate markers are also expressed in PanNET tissues from other studies. Unfortunately, these studies did not sequence non-tumor tissues, so the expression values in non-tumor are only from our study data. Nevertheless, the data shows that these cell surface markers and secretome proteins are expressed in PanNETs and are expressed on a higher level than in non-tumor tissue. Accordingly, they may serve as valid candidates for further testing on protein level in blood plasma of PanNET patients. We've also updated the methods section describing process of comparing our results to other RNA-seq datasets. Please see tracked changes in lines 162 - 169. |
