# Supplementary material for: Transcriptomic profiling of pancreatic neuroendocrine tumors: dysregulation of WNT, MAPK, PI3K, neddylation pathways and potential non-invasive biomarkers
Source: PLoS One. 2025 Jun 16;20(6):e0325672. doi: 10.1371/journal.pone.0325672 (PMC12169574; doi:10.1371/journal.pone.0325672)
Supplement: Attachment — Submitted filename: Response_to_reviewers_auresp_2.docx [file pone.0325672.s015.docx]

| **Reviewer 3** | | |
| --- | --- | --- |
| **Comment** | **Response** |  |
| 1. In Fig. 2A, in the PCA plot, some TAPT and tumor samples look very similar. What could be the reason for this? | Thank you for the comment. I believe this inquiry is related to the two tumor samples that are close to NT tissues. We doubled checked the PCA plot, this time with sample id labels. These two samples are NET22 and NET23 which do not have adjacent non-tumor tissue samples. Therefore, it is rather hard to pinpoint the exact issue why these samples are close to NT samples without additional context from tissue morphology of the tissue section that was used for RNA extraction. Clinically they were both diagnosed G1 tumors which means they retain high level of neuroendocrine differentiation. From technical standpoint it would appear that these two tumor samples which were used for RNA extraction contained significant amount of adjacent non-tumor tissue despite pathologist guided microdissection. We would still like to include these two samples within final results, but we adjusted the results section (please see updated lines 180-182) explaining that there is a possibility that these two tumor samples may contain adjacent non-tumor tissue. |  |
| 2. In Fig. 2B, what value corresponds to which sample? | Thank you for the comment. The plot in fig 2B is a p-value histogramm which shows the distribution of P-values from differential expression analysis comparing all PanNET samples vs. all tumor adjacent pancreatic tissues (TAPT) samples, therefore this is comparision not specific sample data. To better explain this we updated the description within fig2 legend. In general, P-value histogramms are one of the ways to check whether there are any problems with multiple hypothesis tests and to get an overall picture of how significant is the difference between two sample groups. In our case the plot shows that p-values are textbook anti-conservative meaning that there is a statistically significant difference in gene expression between PanNETs and TAPT samples and no alarming issues with the DEseq2 analysis parameters we used. |  |
| 3. The authors mention neddylation, however NEDD8, the genes that authors mention in this pathway, is not one of the top 25 DEGs. | Thank you for comments 3 and 4. NEDD8 was not differentially expressed in our results. We briefly mentioned NEDD8 in discussion section to explain how neddylation system works since the neddylation pathway was the most overrepresented pathway in list of genes differentially expressed in PanNETs. In neddylation, NEDD8 can play a similar role as ubiquitin where NEDD8 ligation alters the stability and function of target protein. NEDD8 also plays an important role in ubiquitination system itself where it is necessery to activate cullin-RING ligases that carry out the ubiquitination process, therefore serving as a molecular switch in the ubiquitination system. When we further explored DEGs related to neddylation using using STRING database we observed that these DEGs form two clusters (S1 Figure). Please see updated sections in lines 239 - 246 in the results section and lines 416 - 423 in the discussion section. According to Gene Ontology (GO) database Cluster 1 DEGs belong to proteasomal complex an ubiquitin ligase complex GO cellular components and proteolysis involved in protein catabolism GO process. The DEGs forming Cluster 2 on the left represent cullin-RING ubiquitin ligase complex and protein ubiquitination process. This indicates that there is perhaps an upregulation of protein degradation via proteasomes that is regulated by neddylation. High proteasome activity has been observed and discussed in other malignancies and has been suggested as one of the therapy targets (29047025). Interestingly there is little infromation about this in PanNETs, possibly due to the fact that RNA-seq based studies in PanNETs are generally rare. Accordingly, this is one of the first reports where the upregulation of proteasomal activity can also be observed in PanNETs warrating further functional investigation as perhaps targeting proteasomal activity could serve as a novel treatment strategy. |  |
| 4. What is neddylation and how is it contributing to the progression of PanNETs? The authors do discuss this briefly in the discussion section, however I believe this requires a more detailed explanation. Does upregulation of neddylation indicate increased proteosome degradation? |  |  |
| 5. In the section 'Transcriptomic changes across different grades of PanNETs', the results should be discussed in more details about the DEGs across different tumor types and their relevance. | Thank you for the suggestion. Indeed, we should have discussed this more in-depth. Although the changes of gene expression between different grades are less pronounced than tumors versus non-tumors, we did find some interesting observations in G3 vs. G1 tumor comparison that we should have discussed within the initial draft of this manuscript. Here, we can observe that several potential oncogenes (LPC2, MYDGF, PLEKHG2, and FOXQ1) are significantly upregulated in G3 NETs compared to G1 NETs. We updated the discussion section discussing these observations. Please see updated lines 449-467 in document with tracked changes. |  |
